# Open Access for the future

**DOI:** 10.1002/elsc.201900152

**Published:** 2019-11-28

**Authors:** An‐Ping Zeng, Thomas Bley, Ralf Takors, Danielle Flemming

We are very happy to announce that *Engineering in Life Sciences* will be a full Gold Open Access journal starting January 1^st^, 2020.

- All papers are now freely accessible for everybody to read, directly upon publication.
- Authors may share their articles with anyone, on any platform or via any communication channel.
- Copyright will remain with the authors under a Creative Commons Attribution (CC‐BY) License which permits use, distribution, and reproduction in any medium (provided the original work is properly cited).
- With Open Access, visibility is significantly increased and research reaches a larger audience; this usually also leads to increased downloads and citations.

The publishing landscape is currently undergoing significant changes. More and more funders and countries now mandate Open Access. They are convinced, similarly to more and more researchers, that science should not be locked behind paywalls, but freely available to read for everyone. So are we! Especially biochemical engineering has a lot to offer and provides numerous solutions for the most driving questions of today for a sustainable future: this science should be easy to find and open to access for anyone.

Of course, the move to Open Access will not affect the editorial process — the scientific evaluation of manuscripts is independent of our publication model. We intend to keep the quality of published papers at the highest scientific level.

Although there are many advantages of Open Access, we understand that there are also potential concerns. To cover the costs of publishing, Article Processing Charges (APC) will be levied for articles that are accepted and published in the journal. However, APCs are covered by many funders, institutions, or national agreements. Please read more https://authorservices.wiley.com/author-resources/Journal-Authors/open-access/affiliation-policies-payments/institutional-funder-payments.html.

The most prominent agreement is the one with “Projekt DEAL” in Germany, covering the costs for Open Access publishing in Wiley journals for the eligible authors from German institutes. Additionally, waivers and discounts are in place, e.g. for authors from developing countries (find the details https://authorservices.wiley.com/open-research/open-access/for-authors/waivers-and-discounts.html).

The transition to Open Access publishing is happening now. We are convinced that this model offers many advantages over traditional publishing and that the free and unlimited access to the content in *Engineering in Life Science* will not only benefit the journal, but strengthen innovations in the field and assist in the quest for a sustainable future and better living conditions for human being.

The ELS editorial team


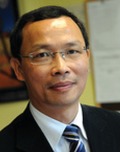


An‐Ping Zeng

(Editor‐in‐Chief)


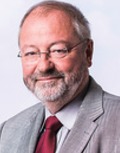


Thomas Bley

(Editor‐in‐Chief)


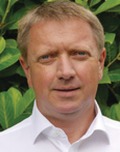


Ralf Takors

(Editor‐in‐Chief)


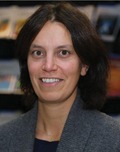


Danielle Flemming

(Managing Editor)

